# Chromosomal Abnormalities in Couples with Primary and Secondary Infertility: Genetic Counseling for Assisted Reproductive Techniques (ART)

**DOI:** 10.18502/jri.v21i4.4331

**Published:** 2020

**Authors:** Subhadra Poornima, Swarnalatha Daram, Rama Krishna Devaki, Hasan Qurratulain

**Affiliations:** 1- Department of Genetics and Molecular Medicine, Kamineni Life Sciences, Moulali, Hyderabad, India; 2- Department of Genetics and Molecular Medicine, Kamineni Hospitals, Hyderabad, India; 3- Department of Biochemistry, Kamineni Academy of Medical Sciences and Research Centre, Hyderabad, India

**Keywords:** Banding, Culturing, Heterochromatin, Infertility, Inversion, Polymorphism, Translocation

## Abstract

**Background:**

World Health Organization estimates that 60–80 million couple worldwide currently suffer from infertility. Recurrent pregnancy loss (RPL) is also another major concern. Chromosomal rearrangements play a crucial role in primary and secondary infertility and RPL. Underlying genetic abnormalities like chromosomal abnormalities contribute to 5–10% of the reproductive failures. The aim of the study was to evaluate the chromosomal abnormalities in infertility and RPL cases to help obstetrician/fertility experts to carry out risk assessment and provide appropriate assisted reproductive techniques for better management of the problem.

**Methods:**

Karyotyping was performed for 414 cases with the history of infertility and RPL over a period of one year. Samples were processed according to procedures of AGT cytogenetic laboratory manual.

**Results:**

Chromosomal abnormalities were observed in 15% of cases. Robertsonian translocation, reciprocal translocation, inversion, derivatives, marker chromosomes, mosaics, aneuploidy and polymorphic variants each contributed 2%, 3%, 3%, 13%, 2%, 10%, 6% and 61%, respectively.

**Conclusion:**

Evaluation of chromosomal abnormalities in couple is warranted prior to planning pregnancy especially for assisted reproductive management cases. Chromosomal analysis can be used as one of the diagnostic tools by OBG/IVF specialists in association with geneticist/genetic counselor for proper reproductive counseling and management.

## Introduction

nfertility is considered as the inability to conceive without using any contraceptives over a period of two years in couples who desire to have a child (1). Infertility can be of two different types: (i) primary infertility which refers to inability to conceive even after unprotected sexual intercourse and (ii) secondary infertility which is inability to continue a pregnancy to term or have a second child. American Society for Reproductive Medicine had defined recurrent pregnancy loss (RPL) as two or more abortions (2). The World Health Organization (WHO) estimates that 60 to 80 million couples worldwide currently suffer from infertility (3). According to global, international and national estimates, the prevalence of RPL and infertility is increasing and efforts have to be reinforced to target the prevention and management aspects (4). Both male and female factors are equally responsible for infertility.

There are many factors contributing to infertility which include hormonal imbalance, anatomical abnormalities, haematological, immunological disorders, infections, environmental factors and the genetic makeup. There are many diagnostic tests available for the assessment and the identification of the underlying causes for infertility and RPL; however, the first genetic assessment in regular clinical practice is a cytogenetic analysis.

Chromosomal rearrangements play a crucial role in infertility and RPL (5). These can be numerical or structural abnormalities. Numerical chromosomal abnormalities mostly involve addition or deletion of a chromosome while structural abnormalities include balanced translocations, inversions, polymorphic variants, heteromorphic and ring chromosomes where the chromosomal structural changes are involved. Presence of chromosomal rearrangements can lead to unequal crossing over during meiosis which results in gametes with unbalanced chromosomes like duplications and deletions (6).

There is an impressive data to support the fact that the complications of the RPL and infertility go beyond the immediate or consecutive pregnancies of patients. Underlying genetic abnormalities like chromosomal abnormalities or single/multi gene mutations or polymorphisms contribute to 5–10% of the reproductive failure. Chromosomal analysis plays a major role in both RPL and infertility case management (7).

The current study emphasizes the importance of evaluating chromosomal abnormalities in individuals and couples with infertility to plan appropriate assisted reproductive technology (ART). Chromosomal analysis can play an important role at least in some couples to prevent birth defects with appropriate prenatal screening and testing. Hence, close interaction between obstetricians, fertility experts and the genetic counselors is important for couples/families to take informed decisions regarding their reproductive life.

The aim of this study was to evaluate the contribution of chromosomal abnormalities in infertility and recurrent pregnancy loss cases and help obstetricians, fertility experts, and genetic counselors for risk evaluation, selecting the most appropriate ART as well as management and treatment.

## Methods

### Sample:

Kamineni Life Sciences houses Credence Diagnostic Centre which is a referral center for genetic testing and counseling. In a period of one year (December, 2017–November, 2018), a total of 414 cases with a history of primary and secondary infertility were referred for karytyping. Three *ml* of heparinised peripheral blood sample was collected from individuals mostly couples for testing.

### Method:

Samples were processed according to AGT cytogenetic laboratory procedures (8). First, blood samples were collected. After exposure to phytohemagglutinin, culturing of lymphocytes was done followed by GTG (G-banding using trypsin and Giemsa) banding with a band level of approximately 400–450 *bp*. Next, karyotypes were described according to ISCN, 2016 (International System for Human Cytogenetic Nomenclature). For every patient, 20 metaphases were analyzed and in case of mosaicism, 50 metaphases were analyzed. Clinical Pathology Accreditation (CPA) guidelines were followed in the procedures and the reporting. The percentage of chromosomal aberrations was calculated in both recurrent pregnancy loss and infertility cases.

## Results

In the present study, 414 individuals were karyotyped including 83 couples and 248 individuals. The percentage of females and males were 51.5%, 48.5% respectively. The percentage of infertility was 48% and RPL cases was 52%. Chromosomal abnormalities accounted for 15% of cases which include reciprocal balanced translocations, robertsonian translocation, derivatives, inversions, polymorphic variants of D and G group chromosomes and the syndromes like Sweyer and Klinefelter. The remaining cases (85%) showed normal chromosomal analysis without any chromosomal abnormalities (Table 1).

**Table 1.** Percentage of normal and abnormal chromosomes in the study

**S. No**	**Classification of chromosomes**	**Frequency**
**I**	Normal karyotypes/no chromosomal abnormalities	352 (85%)
**II**	Chromosomal abnormalities	62 (15%)
**II A.**	Numerical aberrations	4 (1%)
**II B**	Structural aberrations	58 (14%)

### Chromosomal abnormalities:

Major chromosomal abnormalities include numerical aberrations, both reciprocal balanced and robertsonian translocation and mosaics, which were found in 13 patients and the remaining were polymorphic variants in 49 individuals. Robertsonian translocation, reciprocal balanced translocation, inversion, derivatives, marker chromosome, mosaics, aneupliody and polymorphic variants each contributed 2%, 3%, 3%, 13%, 2%, 10%, 6% and 61%, respectively in both infertility and RPL cases (Table 2).

**Table 2.** Percentage of various chromosomal abnormalities in the study

**S. No**	**Type of abnormality**	**Percentage**
**1**	Robertsonian translocation	1 (2%)
**2**	Reciprocal translocation	2 (3%)
**3**	Inversions	2 (3%)
**4**	Derivatives	8 (13%)
**5**	Markers	1 (2%)
**6**	Mosaics	6 (10%)
**7**	Aneploidy	4 (6%)
**8**	Polymorphic variants	38 (61%)

### Klinefelter syndrome:

This is a major numerical chromosomal abnormality. Four males with history of infertility were identified with Klinefelter syndrome.

### Mosaics:

Four female patients with history of recurrent pregnancy loss showed mosaicism. The cases were a 28 year old female and another female patient with BOH also showed mosaicism with 46 XX/46 X+marker chromosome.

### Swyer syndrome:

A 30 year old female with a history of amenorrhea consulted the gynaecologist and was referred for cytogenetic analysis. Ultrasound scan indicated presence of testis. Cytogenetic analysis revealed 46 XY karyotype in the female which indicates Swyer syndrome.

### Robertsonian translocation:

This is one of the main major chromosomal abnormalities. The chromosomes involved in this case were D group 13, 14, 15 and G group 21, 22. A female patient who was 33 years old was identified with 45 XX rob t (14; 22) (p10; p10) with a history of infertility.

### Reciprocal balanced translocation:

Two male patients were identified with balanced chromosomal abnormality. One case with history of infertility was identified with translocation between chromosomes 7 and Y (46 XY, t (7; Y); (q22; p11). Another one was a couple with the history of 4 pregnancy losses in the first trimester. Female partner’s cytogenetic analysis revealed normal 46, XX karyotype and husband’s karyotype was 46 XY, t (3:4); (q13: p 34).

### Derivatives:

This is one of the structural chromosomal abnormalities. In the present study, derivative chromosomes in 6 different patients were identified which included chromosomes 7, 8, 9, 15, 19 and 22. These were in both infertility and RPL cases.

### Inversions:

This is also a structural chromosomal abnormality. The only inversion observed in the study was inv 9. A young female patient of 21 years with a history of three pregnancy losses in the first trimester showed 46 XX inv 9 (p11 q12) and her partner chromosomes were normal without any chromosomal abnormality. Another male patient with history of infertility showed 46 XY inv 9 (p11 q13) and his partner’s karyotype was normal.

### Polymorphic variants:

The most common polymorphic variant other than acrocentric chromosomes was chromosomes 9qh+ and 8 patients were identified with such chromosome. Different satellite regions were observed on chromosomes 13, 14, 15, 21, and 22. Double satellites were observed in 5 patients and satellite regions were confirmed by Ag NOR banding.

## Discussion

Recurrent pregnancy loss or infertility is a devastating experience for couples and also a challenging problem which needs to be addressed. It is mingled with lots of emotional, social and psychological problems for a couple. Currently available data on RPL and infertility is scarce. The possible and the reported risk factors include genetic, uterine anatomical defects, infection, endocrine, immunological factors, clotting disorders, endocrine disorders, infections, advanced maternal age and other general risk factors like alcohol consumption, drugs and uterine injury (9, 10).

The frequency of chromosomal abnormalities among couples indicates that the chromosomal analysis or karyotyping of the couple with reproductive management should be considered for better pregnancies (11). Cytogenetic analysis gives the important genetic information, thus acts as a good diagnostic tool. The breakpoint regions could pave the way for identification of new genes or genes involved in reproductive management and also help in the elucidation of molecular mechanisms underlying the abnormalities.

The present study was planned to assess the chromosomal abnormalities in RPL and infertility cases which helps for risk evaluation and obstetricians/fertility experts can plan an appropriate assisted reproductive technique for treatment and management of couples, which in turn provides control of birth defects.

Chromosomal abnormalities have a major role in RPL and infertility (12). There are mainly two types of chromosomal abnormalities, one is numerical abnormality associated with either the gain or loss of whole chromosomes and other one is structural abnormality which includes the abnormality associated with the structure of the chromosomes (13). The common numerical abnormalities include Klinefelter syndrome, Turner syndrome, *etc*.

Klinefelter cases were observed in the age group of 25–34 years with the history of infertility. These cases were provided with the management in post test counseling sessions like neurodevelopmental and skeletal muscle evaluation and in patients with low testosterone levels, androgen therapy was recommended.

Turner cases were identified with a history of secondary amenorrhea with phenotypic features of short stature, edema of hands or feet, and nail hypoplasia. These cases are sporadic and this is usually seen in all the ethnic groups. Paternal nondisjunction accounts for ~70% of live born Turner syndrome (TS) cases (14, 15). Management aspects were discussed in detail in post test counseling. Audiometric, orthodontic evaluation, thyroid function test, liver enzyme test, blood glucose and lipid test are recommended for TS patients annually. Oestrogen therapy is recommended in case of development of secondary sexual characteristics and also preservation of bone mineral density.

Swyer syndrome is one of the rare forms in cases of primary amenorrhea. In the present study, there was a 30 year old female presented with the history of primary amenorrhea. Clinical history indicated abdominal pain, webbed neck, and cubitus valgus. On examination, no hypopigmented areola in breast was observed and pubic and axillary hair was sparse. Her reports revealed presence of rudimentary uterus and her hormonal profile indicated high levels of FSH. Also, there was an indication of streak gonads. Chromosomal analysis indicated 46, XY karyotpe confirming the diagnosis of Swyer syndrome. In the post test genetic counseling, proband and parents were counseled. The proband had non functional streak gonads leading to inability to produce sex hormones and most of the secondary sexual characters were not developed, hence hormone replacement therapy (HRT) was recommended. These patients can have normal sexual intercourse and they need HRT for development of breast and preventing osteoporosis. Moreover, they can select ART for their problems like using donor oocytes. As there is a high incidence of gonadoblastoma and dysgerminoma, they were informed about gonadectomy as regular surveillance (16).

In the current study, 2% of the chromosomal abnormalities was robertsonian translocation and the incidence in general population was 0.1% and in infertility cases was 1% (17). Individual as a carrier of robertsonian translocation will be phenotypically normal; however, there is a risk of producing unbalanced gametes and therefore unbalanced offspring (18, 19). Carriers of these translocations in recurrent pregnancy loss cases lead to either production of unbalanced gametes causing spontaneous miscarriage of zygote in the first trimester or oogenic disturbances leading to unviable zygotes (20).

Reciprocal balanced translocations were observed in 3% of cases and literature indicates that it leads to miscarriages or the reproductive failure. The length of the translocated chromosomal segment at breakpoints plays a crucial role in the reproductive failure (21). Reciprocal balanced translocations are found to be higher in cases with recurrent pregnancy loss and the prior cytogenetic analysis helps to plan ART or the prenatal testing which resolves the trauma or the psychological stress caused by pregnancy loss to the couple.

Pre implantation genetic testing is also available today. Genetic counselors play an important role in obtaining all the clinical, medical and family history from patients and explaining about the testing and the counseling aspects so that the patients can opt for an appropriate testing after knowing the advantages and the limitations of the tests.

Derivative chromosomes were present in 13% of cases in the current study. High resolution techniques like FISH or array CGH can be performed to know the origin of translocation of particular chromosomes (22, 23). Mosaicism was observed in 10% of cases in this study. Mosaicism is rare; however, the counseling related to this is really a challenging task for a counselor or a geneticist. Sometimes, this remains in somatic cells and sometimes it will be in gonads and causes germinal mosaicism (24).

In reproductive failure cases and recurrent pregnancy loss or secondary infertility cases, heterochromatin variants are the most commonly identified variants and in this chromosome, 9 abnormalities are the major ones (25, 26). In the present study, also 9qh+ is the most common chromosomal variant identified. Earlier studies also showed that the incidence of heterochromatin chromosome 9qh+ is the most common one (27). However, the exact function or the role of this abnormality is not known yet despite the fact that its incidence is higher in infertility and RPL cases (28). Overall, 61% of polymorphic variants are identified in the current study.

## Conclusion

Chromsomal analysis is playing an important role in reproductive management. Evaluation of chromosomal abnormalities in couple is warranted prior to planning pregnancy especially for assisted reproductive techniques. It also helps in appropriate counseling and management and in turn paves the way for controlling and preventing of birth defects. It can be used as one of the diagnostic tools by OBG/IVF specialists in association with geneticist/genetic counselor for proper reproductive counseling and management. Government of India should take an initiative and incorporate this cytogenetic analysis test as a mandate in preconception period in all the health care centers which will be a great asset to the couple either as a means in planning for conception or using assisted reproductive techniques.
